# The Effects of Amine-Carboxyborane Related Derivatives on UMR-106 Bone Metabolism

**DOI:** 10.1155/MBD.1996.31

**Published:** 1996

**Authors:** Margaret E. Murphy, Amy L. Elkins, Robert P. Shrewsbury, Anup Sood, Bernard F. Spielvogel, Iris H. Hall

**Affiliations:** 1 Division of Pharmaceutics School of Pharmacy University of North Carolina Chapel Hill North Carolina 275559-7360 USA; 2 Division of Medicinal Chemistry and Natural Products School of Pharmacy University of North Carolina Chapel Hill North Carolina 275559-7360 USA; 3 Boron Biologicals Inc. 620 Hutton St. Suite 104 Raleigh North Carolina 27606 USA

## Abstract

The amine-carboxyboranes and related derivatives have been shown to be potent anti-inflammatory and anti-osteoporosis agents. Their action in part appears to be mediated by the modulation of cytokines, e.g. TNFα or IL-1. Previous studies have demonstrated that LPS induced macrophages release of TNFα maximally at 60 to 90 min. and IL-1 from 5 to 8 hr. The amine-carboxyboranes reduced significantly the release of these cytokines but also blocked TNFα high affinity binding to UMR-106 receptor at 90 min. at 10 μM, and IL-1 high affinity binding at 5 hr. at 12.5 μM. In addition, the agents suppressed IL-8 binding to CHO K1 high affinity receptor at 24 hr. at 50 μM and IL-2 binding to HuT-8 receptors at 25 μM at 90 min. and 5 hr. Correlation of metabolic events associated with osteoporosis showed that at 90 min., when TNFα receptor binding was reduced by the agents, calcium uptake into UMR-106 cells was reduced at 10 μM as well as the acid and alkaline phosphatases, and the prostaglandin cyclo-oxygenase activities and adhesion of leukocytes and macrophages to UMR-106 cell monolayers. At 5hr. when the agents reduced IL-1 binding to UMR-106 receptors, calcitonin and 1,25-dihydrovitamin D_3_ binding was reduced by the agents as was acid and alkaline phosphatase, and 5′-lipoxygenase activities and white blood cell adhesion. At this time calcium uptake and proline incorporation was increased significantly by the agents. At later times e.g. 18-48 hr. calcium uptake was still increased, and NAG activity was inhibited in the presence of the agents. These effects may be related more to the inhibition of other cytokine receptor binding, e.g. IL-8. Thus, many of the observed metabolic effects of amine-carboxyboranes as antiosteoporosis agents can be correlated with their inhibition of cytokine high affinity binding to target cell receptors.


### THE EFFECTS OF AMINE-CARBOXYBORANE RELATED DERIVATIVES ON UMR-106 BONE METABOLISM

Margaret E. Murphy[1], Amy L. Elkins[2], Robert P. Shrewsbury[1], Anup Sood[3], Bernard F. Spielvogel[3] and Iris H. Hall*[2]

[1] Division of Pharmaceutics, [2] Division of Medicinal Chemistry and Natural Products, School of Pharmacy, University of North Carolina, Chapel Hill, North Carolina 275559-7360, USA
[3] Boron Biologicals, Inc., 620 Hutton St., Suite 104,Raleigh, North Carolina 27606, USA

### Abstract

The amine-carboxyboranes and related derivatives have been shown to be potent anti-inflammatory and anti-osteoporosis agents. Their action in part appears to be mediated by the modulation of cytokines, e.g. TNFα or IL-1. Previous studies have demonstrated that LPS induced macrophages release of TNFα maximally at 60 to 90 min. and IL-1 from 5 to 8 hr. The amine-carboxyboranes reduced significantly the release of these cytokines but also blocked TNFα high affinity binding to UMR-106 receptor at 90 min. at 10 μM, and IL-1 high affinity binding at 5 hr. at 12.5 μM. In addition, the agents suppressed IL-8 binding to CHO K1 high affinity receptor at 24 hr. at 50 μM and IL-2 binding to HuT-8 receptors at 25 μM at 90 min. and 5 hr. Correlation of metabolic events associated with osteoporosis showed that at 90 min., when TNFα receptor binding was reduced by the agents, calcium uptake into UMR-106 cells was reduced at 10 μM as well as the acid and alkaline phosphatases, and the prostaglandin cyclo-oxygenase activities and adhesion of leukocytes and macrophages to UMR-106 cell monolayers. At 5hr. when the agents reduced IL-1 binding to UMR-106 receptors, calcitonin and 1,25-dihydrovitamin $D_3$ binding was reduced by the agents as was acid and alkaline phosphatase, and 5'-lipoxygenase activities and white blood cell adhesion. At this time calcium uptake and proline incorporation was increased significantly by the agents. At later times e.g. 18-48 hr. calcium uptake was still increased, and NAG activity was inhibited in the presence of the agents. These effects may be related more to the inhibition of other cytokine receptor binding, e.g. IL-8. Thus, many of the observed metabolic effects of amine-carboxyboranes as anti-osteoporosis agents can be correlated with their inhibition of cytokine high affinity binding to target cell receptors.

### Introduction

Selected amine-carboxyborane derivatives at 8 mg/kg/day have been shown to prevent osteoporosis and loss of bone mass in rodents[1]. <u>In vitro</u> studies with $CF_1$ mouse pups calvaria and rat UMR-106 osteosarcoma cells have shown that these compounds over 48 hr. reduce the loss of calcium from the cells better than calcitonin and simple boron salts[1]. Indications from the previous <u>in vivo</u> and <u>in vitro</u> studies were that the amine-carboxyboranes effectively lowered TNFα and IL-1 release from macrophages[1,2] which is asociated with a lowering of hydrolytic and proteolytic enzyme activities and secondary chemical effects from prostaglandins and leukotirenes released at the sites of inflammation[3,4] and of bone osteoporosis[1]. The previous studies did not attempt to correlate the incubation time of the drugs with the maximum released of the cytokines into the medium. The present study has been performed in rat UMR-106 bone cells at times, i.e. 90 min. and 5 hr., which have been shown to be the time periods of maximum release of cytokines, TNFα and IL-1, from macrophages. These time points were selected so that we can determine the direct effect of those cytokines on specific biochemical events of bone metabolism. The selected compounds at 8 mg/kg/day orally have been shown in vivo in lactating ovariectomized rats to significantly elevate femur and humerus volume, weight, density and ash weight after 14 days[1]. Also, serum calcium and femur calcium levels were elevated whereas hydroxyproline levels were reduced in rodents by the amine-carboxyboranes.

### Methods and Materials

The amine-carboxyborane derivatives were previously synthesized and the chemical and physical characteristics reported[1]: Compound 1 $(C_6H_5)_3PBH_2COOH$, Compound 2 $C_{16}H_{33}N(CH_3)_2BH_2COOH$ and Compound 3 $(CH_3)_3NBH_2COOCH_3$. TNFα, IL-1, IL-2, IL-8 and the standards were purchased from Sigma Chemical Co.

Cell Maintenance

Rat UMR-106 osteosarcoma cells (DMEM + 10% fetal calf serum (FCS) + penicillin/streptomycin [P/S]) and IC-21 mouse macrophages (RPMI 1640 + 10% FCS + P/S) were grown to confluency[1]. Mouse macrophages (J774 A) were maintained in DMEM + 15% FCS + P/S; human peripheral leukocytes (RPMI 1788) were maintained in RPMI 1640 + 15% FCS + P/S. Chinese hamster ovaian (CHO) K1 cells were maintained in EMEM + 10% FCS + nonessential amino acids + P/S. HuT-8 cells human cutaneous T cell lymphoma were maintained in RPMI + 10% FCS + gentamycin/kanamycin.

High Affinity Binding to Receptors on UMR-106 Cells

Two μCi of $^{125}$I-TNFα (human recombinant , 30 mCi/μg New England Nuclear) or $^{125}$I-IL-1 (70-120 μCi/μg,New England Nuclear) was added to confluent UMR-106 cells for 30 to 120 min with the amine-carboxyboranes from 10 to 50 μM [5]. $^{125}$IL-8 [2Ci/mol, Amersham] was added to confluent CHO K1 cells for 5 to 24 hr. and $^{125}$IL-2 [20-50 μCi/ug, New England Nuclear] was added to confluent HuT-8 cells from 5 to 48 hr with drugs. All of these cells have high affinity receptor within their plasma membrane for the specific cytokine. After the allotted time period, the medium was decanted gently and the cells were washed 6 X in cold isotonic PBS, pH 7.2. The cells were taken up in 0.1N NaOH and aliquots were counted. $^{125}$I-Calitonin (human, 2000 Ci/nmole, Amersham) or $^3$H-1,25-dihyroxyvitamine $D_3$ (155-175 Ci/mmole, New England Nuclear) 10 μCi were added to confluent UMR-106 cells with drugs from 10 to 100 μM and incubated 90 min. or 5 hr. Cells were washed repeatedly in PBS and take-up in 0.1 N NaOH. In the case of 1,25-dihydro-vitamin $D_3$, the nuclei preparation was obtained. The aliquots were counted in a Pachard Beta counter corrected for quenching, and non-specific binding of isotopes.

$^{45}$Ca Uptake and $^3$H Proline Incorporation into Collagen of UMR-106 Cells

$^{45}CaCl_2$ (0.2 mCi, New England Nuclear) was added to confluent UMR 106, The medium was decanted and the cells were washed 4 times with PBS, pH

7.2 [6,7]. The cells were taken up in 0.1N NaOH and counted [24,25]. Rat UMR-106 cells (95% confluent) were incubated with 1 μCi of 2,3,4,5-$^3$H-proline (102 Ci/mmol., New England Nuclear) and drugs. After 24 hr, the medium was discarded and the cells were washed repeatedly with PBS. The cells were harvested in 0.1N NaOH. After treatment with 20% TCA, cells were centrifuged at 3500 g X 5 min. The supernatant was discarded and the pellet was suspended in 1 ml of 50 mM Tris + 5 mM $CaCl_2$ + 20 μl of collagenase (10 mg/ml buffer, Sigma Chemical Co. [St. Louis, MO]) and incubated for 2 hr at 37°C. Tannic acid:TCA (5%)[1:1] solution was added and incubated for 15 min at room temperature followed by centrifugation to separate the collagen from non-collagen protein[8].

Lysosomal Enzymes
UMR-106 cells [$10^8$] were grown to confluency and incubated for 90 min. or 5 hr. with drugs at 12.5, 50 and 100 μM. Acid and alkaline phosphatase activities were determined using 0.1 M β-glycerolphosphate in 0.1 M acetate buffer, pH 5.0 and 8.5, respectively[1-3]. Total enzyme was released with Triton X-100. The reaction was terminated with 10% TCA and then the solution was centrifuged at 3500 g for 10 min. The inorganic phosphate in the supernatant was determined spectrophotometrically at 800 nm by the method of Chen et al.[9]. N-Acetylglucosaminidase [NAG] activity was determined by the method Tulberg-Reinert and Hetti[10]. UMR-106 cells were incubated with drugs at 12.5, 50 and 100 μM for 90min. and 5 hr. in the presence of 10 μg/ml LPS (Salmonella abortus equi). The medium[50 μl] was incubated with 7.5 mM *p*-nitrophenyl-N-acetyl-β-D-glucosaminide in glycine buffer, pH 5.0 in 96-well microplates. The reaction was stopped by the addition of glycine/EDTA buffer [50mM:5mM]. The concentration of *p*-nitrophenol released was measured at 405 nm using a microplate reader [Thermomax, Molecular Devices Corp.].

Prostaglandin Synthetase Activity
The incubation procedures of Tomlinson et al.[11] and Glatt et al.[12] were used to determine prostaglandin formation from $^3$H(N)-arachidonic acid (100 Ci/mmole) and UMR-106 cells ($10^6$). After 1 hr, the reaction was terminated with 2N HCl and the mixture was extracted with ether and evaporated. The residue was dissolved in ethyl acetate and applied to silica gel TLC plates which were eluted with chloroform, methanol, water and acetic acid (90:8:1:0.8). The plates were developed with iodine vapor and the area appropriate to the prostaglandin standards was scraped and counted [12,13]. The dpm in each area was calculated as percent of the total dpm applied to the plate.

5'-Lipoxygenase Assay
UMR-106 cells were incubated for 30 min with phosphate buffer (pH 7.2) containing 0.6 mM $CaCl_2$, and 1.0 mM $MgCl_2$, 10 mg Calcium Ionophore A23187 and 1 mCi $^{14}$C-arachidonic acid (100 Ci/mmol). The reaction was terminated with 2 volumes of EtOAc:$CH_2Cl_2$ containing 12 mg cold arachidonic acid. The organic phase was evaporated to a residue which was applied to silica gel plates. The plates were eluted with chloroform:methanol:water:acetic acid (90:8:1:0.8). The 5-HETE area corresponding to the standard was scraped and counted[13,14].

Cell Adhesion to UMR-106 Cells
RPMI 1788 leukocytes or J774.A1 mouse macrophages [$10^8$] cells were incubated with 10 μCi of $^3$H-thymidine (New England Nuclear, 78.3 Ci/mmole) for 3 hr. and growth medium[15,16]. The cells were centrifuged at 1000 rpm for 5 min, rinsed in isotonic PBS, pH 7.2 and resuspended in fresh medium. Aliquots of labeled cells were added to confluent UMR-106 cells and the drugs were added at 25, 50 or 100 μM and incubated for 1.5, 2 and 5 hr. The medium was decanted and the UMR-106 cells were washed repeatedly with PBS. The cells were taken up in 0.1N NaOH and aliquots were counted.

### Results

Preliminary studies have demonstrated that IC-21 macrophages when incubated with LPS [E. coli] at 10μg/ml release TNFα from 60-120 min with 90 min. being the maximum time of release. IL-1 under similar conditions was released from $P388_{D1}$ with the peak elevation being at 5 hr. The IL-1 levels remainder elevated through 8 hr. and then fell slowly back to normal levels. Thus, the following biochemical parameters were measured in the presence of the amine-carboxyboranes at these peak times of the two cytokines.

Incubation of $^{125}$I-TNFα with UMR-106 cells showed peak high affinity binding at 90 min. [Table 1a and b]. Compound 1 at 10 μM at 60 min caused at 34% reduction in the high affinity binding of TNFα whereas Compounds 2 and 3 caused 18% and 33% reduction in binding at 90 min. When higher concentration of the drugs were employed at 90 min incubation, elevated high affinity binding of TNFα to UMR-106 receptors was observed [Table 1b].

**Table 1a. The Effect of Amine-carboxyboranes at 10 μM on TNFα High Affinity Receptor Binding on UMR-106 Cells**

**% of Control** *(N=6)*

| Time (minutes) | Control | COMPOUND #1 | COMPOUND #2 | COMPOUND #3 |
|---|---|---|---|---|
| 30 | 100±9[a] | 86±8 | 121±8 | 98±7 |
| 60 | 100±8[b] | 66±3* | 84±6 | 81±5* |
| 90 | 100±13[c] | 94±9 | 82±9 | 67±6* |
| 120 | 100±14[d] | 96±8 | 89±9 | 84±9 |

Control Values: [a] 1784 CPM/mg protein; [b] 2224 CPM/mg protein; [c] 3273 CPM/mg protein; [d] 1669 CPM/mg protein; * $p<0.001$

**Table 1b. The Effect of Amine-carboxyboranes on TNFα High Affinity Receptor Binding on UMR-106 Cells After 90 Minutes**

**% of Control** *(N=6)*

| Conc. (μM) | COMPOUND #1 | COMPOUND #2 | COMPOUND #3 |
|---|---|---|---|
| 12.5 | 129±9* | 116±13** | 153±17* |
| 25 | 96±4 | 164±8* | 160±11* |
| 50 | 108±11 | 147±15* | 217±10* |

Control = 100±12% (1.84 CPM/mg protein); * $p<0.0001$** $p<0.05$

$^{125}$I-IL-1 high affinity binding to UMR-106 cells peaked at 5 hr. (815 cpm/mg protein) and was still high at 8 hr. (742 cpm/mg protein) [Table 2a]. When the agents were incubated with labeled IL-1, at 5 hr. the concentration of 12.5 μM afforded the maximum reduction for all three agents [Table 2b]. Concentrations of 25 and 50 μM of the agents were less effective in reducing the binding of IL-1 to its receptor. $^{125}$I-IL-8 high affinity binding to CHO-K1 membrane receptors was maximum at 24 hr. This binding was suppressed approximately 46-49% by all three agents at 50 μM [data not shown] and $^{125}$-IL-2 high affinity binding to HuT-8 cells receptors was suppressed at 25 and 50 μM of the agents at 90 min and at 5 hr. [Tables 2c and d].

**Table 2a. The Effect of Amine-carboxyboranes at 10 μM on IL-1 High Affinity Receptor Binding on UMR-106 Cells**

% of Control *(N=6)*

| Time (hours) | Control | Compound #1 | Compound #2 | Compound# 3 |
|---|---|---|---|---|
| 1.5 | 100±6[a] | 184±21* | 107±16 | 152±5* |
| 5 | 100±6[b] | 105±8 | 71±14* | 67±2* |
| 8 | 100±8[c] | 123±5* | 87±9 | 124±9* |
| 24 | 100±6[d] | 94±5 | 93±14 | 114±12 |
| 30 | 100±8[e] | 109±8 | 111±8 | 98±9 |
| 48 | 100±5[f] | 93±6 | 74±3* | 109±4* |

Control Values: [a] 653 CPM/mg protein; [b] 815 CPM/mg protein; [c] 742 CPM/mg protein; [d] 274 CPM/mg protein; [e] 270 CPM/mg protein; [f] 231 CPM/mg protein; *p<0.01

$^{125}$I-Calcitonin high affinity binding to UMR-106 cell receptors was the highest from 60 to 120 min. and then declined. Compound 1 at 10 μM caused a 38% reduction of calcitonin binding at 60 min. and a 51% reduction at 8 hr. [Table 3]. Compound 2 at 10 μM caused a 45% and 64% reduction at 5 and 8 hr., respectively and Compound 3 caused a 45% reduction at 5 hr.

**Table 2b. The Effect of Amine-carboxyboranes on IL-1 High Affinity Receptor Binding on UMR-106 Cells After 5 Hours**

% of Control *(N=6)*

| Conc. (μM) | COMPOUND #1 | COMPOUND #2 | COMPOUND #3 |
|---|---|---|---|
| **12.5** | 37±3* | 64±5* | 47±8* |
| **25** | 59±6* | 89±9 | 68±6* |
| **50** | 68±8* | 75±5* | 82±7* |

Control = 100±6% (581.2 CPM/mg protein);* p<0.0001

**Table 2c. The Effect of Amine-carboxyboranes on IL-2 High Affinity Binding to Receptors on HuT-8 Cells After 90 Minutes**

% of Control (N=6)

| Conc. (μM) | COMPOUND #1 | COMPOUND #2 | COMPOUND #3 |
|---|---|---|---|
| 12.5 | 98±6 | 93±5 | 96±4 |
| 25 | 32±3* | 56±2* | 31±3* |
| 50 | 0±1 | 31±5* | 0+2 |

Control = 100±5% (1.517 CPM/mg protein); * p = 0.001.

**Table 2d. The Effect of Amine-carboxyboranes on IL-2 High Affinity Binding to Receptors on HuT-8 Cells After 5 Hours**

% of Control (N=6)

| Concentration μM) | COMPOUND #1 | COMPOUND #2 | COMPOUND #3 |
|---|---|---|---|
| 12.5 | 96±5 | 57±6* | 91±6 |
| 25 | 50±4* | 52±5* | 32±4* |
| 50 | 32±3* | 51±5* | 0±2 |

Control = 100±4% (1.06 CPM/mg protein); *p = 0.001

**Table 3. The Effect of Amine-carboxyboranes at 10 μM on Calcitonin High Affinity Binding to Receptors on UMR-106 Cells**

% of Control (N=8)

| Time (hours) | Control | Compound #1 | Compound #2 | Compound #3 |
|---|---|---|---|---|
| 1 | 100±11[a] | 62±5* | 105±8 | 105±10 |
| 1.5 | 100±14[b] | 78±25b | 93±11 | 85±11 |
| 2 | 100±8[c] | 83±16 | 80±16 | 74±14* |
| 5 | 100±8[d] | 98±2 | 55±12* | 55±9* |
| 8 | 100±7[e] | 49±6* | 36±8* | 88±14 |
| 24 | 100±9[f] | 97±4 | 88±13*** | 87±19 |
| 30 | 100±9[g] | 96±12 | 122±6 | 89±19 |
| 42 | 100±16[h] | 80±15* | 83±15 | 120±17 |
| 48 | 100±7[i] | 127±16* | 78±23 | 126±16 |

Control Values: [a]1954 CPM/mg protein; [b]2020 CPM/mg protein; [c]1940CPM/mg protein; [d]1753 CPM/mg protein; [e]1520 CPM/mg protein; [f] 1793 CPM/mg protein; [g]933 CPM/mg protein; [h] 826 CPM/mg protein; [i] 1447 CPM/mg protein; *p<0.001.

---

1,25-Dihydroxyvitamin-D high affinity binding to UMR-106 nuclear receptors was elevated by Compound 1 at 10 µM at 8 hr. and Compound 3 caused significant elevations at 8, 24 and 30 hr. [Table 4a]. Higher concentrations of the agents had little effects on the elevations and in fact at 50 µM at 5 hrs. the Compounds actually caused a significant reduction in binding [Table 4b and 4c]. TNFα at 10 and 100 ng/ml at 90 min caused at 26 and 20% elevation in binding of 1,25-dihydrovitamin-$D_3$ to its nuclear receptor in UMR-106 cells.

---

**Table 4a. The Effect of Amine-carboxyboranes at 10 µM on DHD High Affinity Binding to Nuclear Receptors in UMR-106 Cells**

% of Control (N=8)

| Time (hours) | Control | Compound #1 | Compound #2 | Compound #3 |
|---|---|---|---|---|
| 1.5 | 100±5[a] | 118±17 | 118±5 | 128±9* |
| 5 | 100±9[b] | 117±10 | 114±11 | 98±19 |
| 8 | 100±8[c] | 169±6* | 115±10 | 166±16* |
| 24 | 100±6[d] | 129±16 | 105±23 | 223±11* |
| 30 | 100±6[e] | 122±9 | 110±19 | 151±17* |
| 48 | 100±8[f] | 111±13 | 119±15 | 102±7 |

Control Values: [a] 2143 DPM/mg protein; [b]2475 DPM/mg protein; [c]1812 DPM/mg protein; [d] 1333 DPM/mg protein; [e] 1393 DPM/mg protein; [f] 1355 DPM/mg protein; *p<0.0001.

---

Calcium uptake by UMR-106 cells peaked at 90 min. and then gradually declined over the next 48 hr. Incubation of the drug at 10 µM [Table 5a] indicated that calcium uptake was markedly reduced at 90 min. and that TNFα at 10 ng/ml also caused a significant reduction of 56% whereas at 100 ng/ml TNFα caused only a 6% reduction at 90 min.[data not shown].

**Table 4b. The Effect of Amine-carboxyboranes at 100 μM on DHD High Affinity Binding to Nuclear Receptors in UMR-106 Cells**

% of Control (N=8)

| Time (hours) | Control | Compound #1 | Compound #2 | Compound#3 |
|---|---|---|---|---|
| 1 | 100±17[a] | 105±9 | 101±6 | 108±6 |
| 1.5 | 100±5[b] | 83±13 | 86±8 | 94±15 |
| 2 | 100±19[c] | 75±13* | 90±18 | 115±8 |
| 5 | 100±9[d] | 115±23 | 103±8 | 130±22** |
| 8 | 100±8[e] | 72±26** | 95±6 | 106±16 |
| 24 | 100±6[f] | 111±11 | 103±13 | 105±3 |
| 42 | 100±14[g] | 111±18 | 96±5 | 99±11 |
| 48 | 100±8[h] | 128±29 | 99±11 | 110±19 |

Control Values: [a] 1329 DPM/mg protein; [b] 2143 DPM/mg protein; [c] 2200 DPM/mg protein; [d] 2475 DPM/mg protein; [e] 1812 DPM/mg protein; [f] 1333 DPM/mg protein; [g] 1302 CPM/mg protein; [h] 1355 CPM/mg protein; * p<0.0001; ** p<0.025.

**Table 4c. The Effect of Amine-carboxyboranes on DHD High Affinity Binding to Nuclear Receptors in UMR-106 Cells After 5 Hours**

% of Control (N=8)

| Concentration (μM) | COMPOUND #1 | COMPOUND #2 | COMPOUND #3 |
|---|---|---|---|
| 10 | 117±10 | 114±11 | 98±19 |
| 12.5 | 73±19* | 91±8 | 87±17 |
| 25 | 70±23* | 80±17** | 74±13* |
| 50 | 53±16* | 77±14* | 53±23* |
| 100 | 115±23 | 103±8 | 130±22 |

Control = 100±13% (3246 DPM/mg protein); * p<0.0001; ** p<0.005.

Reduction of calcium uptake by UMR-106 cells peaked at 90 min. and then gradually the agents at 10 μM reversed the suppression of calcium influx over time. At 8 and 12 hr. the agents caused a significant elevation in calcium uptake but from 18 to 48 hr. The influx of calcium into bone cells was reduced. Calcium uptake into UMR-106 cells after a 5hr. incubation, fell at 10 μM but at higher concentration of the agents there was an elevation in calcium uptake. Compound 1 doubled the calcium uptake at 25 μM but fell at 50 μM [Table 5b]. Compound 2 caused a doubling of calcium uptake at 5 hr. at 25 and 50 μM . Compound 3 was less effective and showed 9% and 12% increases at 25 and 50 μM, respectively.

---

**Table 5a. The Effect of Amine-carboxyboranes at 10 μM on Calcium uptake into UMR-106 Cells**

<u>% of Control</u> *(N=8)*

| Time (hours) | Control | Compound #1 | Comound #2 | Compound #3 |
|---|---|---|---|---|
| 1 | 100±14[a] | 78±17* | 89±17 | 97±10 |
| 1.5 | 100±8[b] | 39±7* | 31±17* | 20±10* |
| 2 | 100±8[c] | 64±16* | 64±25* | 78±7* |
| 5 | 100±18[d] | 93±16 | 86±18 | 88±19 |
| 8 | 100±19[e] | 154±40* | 230±39* | 230±20* |
| 12 | 100±29[f] | 129±34 | 110±32 | 132±24*** |
| 18 | 100±19[g] | 63±24* | 74±12* | 68±19* |
| 24 | 100±12[h] | 53±6* | 55±9* | 47±4* |
| 36 | 100±7[i] | 47±12* | 57±16* | 49±9* |
| 48 | 100±24[j] | 40±16* | 59±14* | 53±19* |

Control Values:[a] [g] 6522 CPM/mg protein:[f] [c] 873 CPM/mg protein; [b] [h] 11825 CPM/mg protein; [d] 2800 CPM/mg protein;[i] 7900 CPM/mg protein;[e] 5925 CPM/mg protein; 6691 CPM/mg protein; 5751 CPM/mg protein; 5385 CPM/mg protein;[j] 2587 CPM/mg protein;* $p<0.0001$; ** $p<0.01$; *** $p<0.05$

---

**Table 5b. The Effect of Amine-carboxyboranes on Calcium Uptake into UMR-106 Cells After 5 Hours**

<u>% of Control</u> *(N=8)*

| Concentration (μM) | COMPOUND #1 | COMPOUND #2 | COMPOUND #3 |
|---|---|---|---|
| 10 | 93±16 | 86±18**** | 88±19 |
| 12.5 | 111±10*** | 123±2* | 109±10**** |
| 25 | 193±10* | 207±8* | 112±9** |
| 50 | 84±8* | 214±10* | 65±6* |

Control=100±9 (14,500 CPM/mg protein);* $p<0.0001$; ** $p<0.01$; *** $p<0.025$;****$p<0.05$

---

Proline incorporation into collagen of UMR-106 cells followed a time dependent effect over 48 hr. [Table 6a]. The agents at 10 μM caused a significant increase in proline incorporation at 5 and 8 hr. but not at 24 and 48 hr. Proline incorporation into non-collagen protein of UMR-106 cells was elevated only at 8 hr. by Compounds <u>1</u> and <u>2</u> [Table 6b]. Compound <u>2</u> at 12.5, 25 and 50 μM markedly elevated proline incorporation whereas Compounds <u>1</u> and <u>3</u> had no effect or actually reduced incorporation at 12.5 μM.[Table 6c]. Non-collagen incorporation of proline was not affected by the higher concentrations of any of the agents [Table 6d]. TNFα at 100 ng/ml at 5 hr. caused a 49% increase of proline incorporation into collagen but at 10 ng/ml had no affect at 90 min. or 5 hr. nor was there any effect on the non-collagen incorporation.

**Table 6a. The Effect of Amine-carboxyboranes at 10 μM on Proline Incorporation into Collagen in UMR-106 Cells**

% of Control (N=6)

| Time (hours) | Control | COMPOUND #1 | COMPOUND #2 | COMPOUND #3 |
|---|---|---|---|---|
| 1.5 | 100±11[a] | 115±8** | 98±9 | 116±10** |
| 5 | 100±14[b] | 207±7* | 142±21* | 203±11* |
| 8 | 100±8[c] | 544±4* | 271±27* | 363±27* |
| 24 | 100±8[d] | 69±10* | 93±5*** | 84±7* |
| 48 | 100±7[e] | 57±6* | 101±21 | 69±24* |

Control values:a 4844 DPM/ml; b 5113 DPM/ml; c 6986 DPM/ml; d 9396 DPM/ml; e 13595 DPM/ml; * p<0.0001;** p<0.01;***p<0.025

UMR-106 NAG hydrolytic activity was not inhibited by the amine-carboxyboranes at 90 min. or 5 hr. from 10 to 100 μM. Time periods of 24-72 hr. were found to be needed to observed the inhibition by the agents. Compound 3 caused 25% and 31% at 24 and 48 hr., respectively at 10 μM.[Table 7].

**Table 6b. The Effect of Amine-carboxyboranes at 10 μM on Proline Incorporation into Non-Collagen in UMR-106 Cells**

% of Control (N=6)

| Time (hrs) | Control | COMPOUND #1 | COMPOUND #2 | COMPOUND #3 |
|---|---|---|---|---|
| 1.5 | 100±2[a] | 114±5* | 97±2*** | 97±2*** |
| 5 | 100±11[b] | 118±5* | 96±9 | 101±9 |
| 8 | 100±16[c] | 144±30** | 200±6* | 110±9 |
| 24 | 100±5[d] | 72±8*** | 58±4* | 45±3* |

Control Values:[a] 289 DPM/ml; [b] 406 DPM/ml; [c] 804 DPM/ml;[d] 1721 DPM/ml; [e] 1078 DPM/ml; * p<0.0001;** p<0.005;*** p<0.05

**Table 6c The Effect of Amine-carboxyboranes on Proline Incorporation into Collagen in UMR-106 Cells After 5 Hours**

% of Control (N=6)

| Concentration (μM) | COMPOUND #1 | COMPOUND #2 | COMPOUND #3 |
|---|---|---|---|
| 12.5 | 62±13* | 279±15* | 63±5* |
| 25 | 93±9 | 229±13* | 81±7* |
| 50 | 95±25 | 199±18* | 77±16** |

Control=100±12% (2380 DPM/ml);* p<0.0001;**p<0.005

Compound 2 caused a 36% reduction and Compound 1 caused a 25% reduction at 10 µM after 48 hr. The acid and akaline phosphatase activities in UMR-106 cells were significantly reduced in the presence of the amine-carboxyboranes from 12.5 to 50 µM at 90 min., 5 and 18 hrs. [Tables 8a-c and 9a-c].

**Table 6d. The Effect of Amine-carboxyboranes on Proline Incorporation into Non-Collagen in UMR-106 After 5 Hours**
**% of Control** *(N=6)*

| Concentration (µM) | COMPOUND #1 | COMPOUND #2 | COMPOUND #3 |
|---|---|---|---|
| 12.5 | 108±10*** | 124±8* | 120±4* |
| 25 | 86±8** | 94±4*** | 84±3* |
| 50 | 96±2 | 84±6 | 108±8*** |

Control = 100±10%(1000 DPM/ml); * p,0.000.1; **p,0.01; ***p,0.05.

**Table 7. The Effect of Amine-carboxyboranes at 1 or 10 µM on NAG Activity in UMR-106 Cells**
**% of Control** *(N=8)*

| Time (hours) | Control | CMPD #1 1µM | CMPD #1 10µM | CMPD #2 1µM | CMPD #2 10µM | CMPD #3 1µM | CMPD #3 10µM |
|---|---|---|---|---|---|---|---|
| 24 | 100±5[a] | 125±14* | 130±10* | 105±6 | 104±4 | 93±5* | 75±13* |
| 48 | 100±6[b] | 81±6* | 75±12* | 86±4* | 64±4* | 81±5* | 69±5* |
| 72 | 100±4[c] | 88±5* | 103±8 | 114±5* | 87±5* | 95±5 | 88±6* |

Control Values: [a] 12 nmoles *p*-nitrophenol released/ml medium; [b] 39 nmoles p-nitrophenol released/ml medium; [c] 40 nmoles *p*-nitrophenol released/ml mediu; * p<0.0001

**Table 8a. The Effect of Amine-carboxyboranes on Acid Phosphatase Activity in UMR-106 Cells After 90 Minutes**
**% of Control** *(N=10)*

| Concentration (µM) | COMPOUND #1 | COMPOUND #2 | COMPOUND #3 |
|---|---|---|---|
| 12.5 | 51±3* | 56±5* | 51±5* |
| 25 | 28±4* | 30±7* | 30±3* |
| 50 | 26±3* | 20±3* | 25±4* |

Control = 100±7% (100 mg $P_i$/mg protein/90 minutes); * p<0.0001

UMR-106 prostaglandin cyclo-oxygenase activity was suppressed significantly by the agents at 90 min. and 5 hr. [Table 10a and b].

**Table 8b. The Effect of Amine-carboxyboranes on Acid Phosphatase Activity in UMR-106 Cells After 5 Hours**

% of Control (N=10)

| Concentration (μM) | COMPOUND #1 | COMPOUND #2 | COMPOUND #3 |
|---|---|---|---|
| 12.5 | 82±6* | 63±5* | 57±4* |
| 25 | 50±3* | 62±6* | 39±6* |
| 50 | 23±5* | 56±5* | 53±6* |

Control = 100±6% (200 mg $P_i$/mg protein/5 hours);* p<0.0001

**Table 8c Effect of Amine-carboxyboranes on Acid Phosphatase Activity in UMR-106 Cells After 18 Hours**

% of Control (N=10)

| Concentration (μM) | COMPOUND #1 | COMPOUND #2 | COMPOUND #3 |
|---|---|---|---|
| 12.5 | 80±7* | 89±9* | 86±8 |
| 25 | 61±7* | 70±8* | 51±5* |
| 50 | 43±5* | 43±6* | 16±4* |

Control = 100±9% (716 mg $P_i$/mg protein/18 hours);* p<0.0001;

**Table 9a. The Effect of Amine-carboxyboranes on Alkaline Phosphatase Activity in UMR-106 Cells After 90 Minutes**

% of Control N=10)

| Concentration (μM) | COMPOUND #1 | COMPOUND #2 | COMPOUND #3 |
|---|---|---|---|
| 12.5 | 76±6* | 69±8* | 67±9* |
| 25 | 57±7* | 51±5* | 56±4* |
| 50 | 54±5* | 45±5* | 44±9* |

Control = 100±9% (334 mg $P_i$/mg protein/90 minutes); * p<0.0001

**Table 9b. The Effect of Amine-carboxyboranes on Alkaline Phosphatase Activity in UMR-106 Cells After 5 Hours**

% of Control (N=10)

| Concentration (μM) | COMPOUND #1 | COMPOUND #2 | COMPOUND #3 |
|---|---|---|---|
| 12.5 | 83±8 | 80±9 | 81±7 |
| 25 | 78±6* | 48±6* | 62±6* |
| 50 | 67±7* | 46±7* | 56±5* |

Control = 100±7% (700 mg $P_i$/mg protein/5 hours); * p<0.0001

---

**Table 9c. The Effect of Amine-carboxyboranes on Alkaline Phosphatase Activity in UMR-106 Cells After 18 Hours**

| Concentration (μM) | % of Control (N=10) | | |
|---|---|---|---|
| | COMPOUND #1 | COMPOUND #2 | COMPOUND #3 |
| 12.5 | 69±7* | 69±8* | 59±3* |
| 25 | 63±5* | 54±5* | 48±5* |
| 50 | 47±8* | 50±4* | 41±3* |

Control = 100±8% (1223 mg $P_i$/mg protein/18 hours); * $p<0.0001$

---

**Table 10a Effect of Amine-carboxyboranes on Prostaglandin Cyclo-oxygenase Activity in UMR-106 Cells After 90 Minutes**

| Concentration (μM) | % of Control (N=6) | | |
|---|---|---|---|
| | COMPOUND #1 | COMPOUND #2 | COMPOUND #3 |
| 12.5 | 36±7* | 87±5 | 52±6* |
| 25 | 50±4* | 54±4* | 43±7* |
| 50 | 35±5* | 41±5* | 38±4* |

Control = 100±5% (16,134 DPM/mg protein)

---

**Table 10b. The Effect of Amine-carboxyboranes on Prostaglandin Cyclo-oxygenase Activity in UMR-106 Cells After 5 Hours**

| Concentration (μM) | % of Control (N=6) | | |
|---|---|---|---|
| | COMPOUND #1 | COMPOUND #2 | COMPOUND #3 |
| 12.5 | 90±7 | 52±4* | 92±6 |
| 25 | 82±6 | 67±4* | 82±6 |
| 50 | 79±6* | 78±5* | 76±5* |

Control = 100±5% (14,480 DPM/mg protein); * $p = 0.001$.

---

**Table 11a. The Effect of Amine-carboxyboranes on 5'-Lipoxygenase Activity in UMR-106 Cells After 90 Minutes**

| Concentration (μM) | % of Control (N=6) | | |
|---|---|---|---|
| | COMPOUND #1 | COMPOUND #2 | COMPOUND #3 |
| 12.5 | 112±6 | 93±6 | 105±8 |
| 25 | 91±7 | 74±7* | 97±7 |
| 50 | 85±4* | 63±5* | 94±6 |

Control = 100±8 % (2606 DPM/mg protein); * $p<0.0001$.

---

Whereas, UMR-106 5'-lipoxygenase activity was not inhibited significantly by the agents at 90 min with the exception of Compound 2

at 25 and 50 μM. At 5 hr. all of the compounds at 50 μM caused inhibition of 5'-lipoxygenase activity [Table 11a and b].

---

**Table 11b. The Effect of Amine-carboxyboranes on 5'-Lipoxygenase Activity in UMR-106 Cells After 5 Hours**

| Concentration (μM) | % of Control (N=6) COMPOUND #1 | COMPOUND #2 | COMPOUND #3 |
|---|---|---|---|
| 12.5 | 118±9 | 107±6 | 93±7 |
| 25 | 83±8 | 90±4 | 86±7 |
| 50 | 66±7* | 66±5* | 83±6 |

Control = 100±8% (3798 DPM/mg protein); * $p<0.0001$;** $p<0.005$;*** $p<0.05$

---

Leukocyte and macrophage adhesion to confluent UMR-106 cells showed that leukocytes adhesion was maximally reduced by the compounds at 50 μM at 90 min. while at 5 and 8 hrs., there was partial reduction of the adhesion [Table 12a]. Longer period of times showed tha no reduction in leukocyte adhesion occured. Macrophage adhesion to UMR-106 cells was accelerated at lower concentrations of 5-25 μM at 90 min. while at 50 μ M, all three compounds caused at significant reduction in adhesion. At 2 and 5 hr., the agents afforded a concentration dependent reduction of macrophages adhesion to confluent bone cells [Table 12b-d].

---

**Table 12a. The Effect of Amine-carboxyboranes at 50 μM on $^{3}$H-RPMI-1788 Human Leukocyte Adhesion to UMR-106 Cells**

| Time (hours) | Control | % of Control (N=6) COMPOUND #1 | COMPOUND #2 | COMPOUND #3 |
|---|---|---|---|---|
| 1.5 | 100±7[a] | 60±5* | 76±4* | 62±5* |
| 5 | 100±7[b] | 89±6 | 75±5* | 83±6 |
| 8 | 100±7[c] | 137±7* | 68±6* | 67±5* |
| 18 | 100±7[d] | 104±6 | 106±7 | 105±6 |
| 24 | 100±7[e] | 97±8 | 116±7 | 108±7 |

Control Counts: [a] 92,000 DPM/mg protein; [b] 90,800 DPM/mg protein; [c] 217,200 DPM/mg protein; [d] 31,000 DPM/mg protein; [e] 48,600 DPM/mg protein*$p<0.0001$

---

**Table 12b. The Effect of Amine-carboxyboranes on J774.A1 Mouse Macrophage Adhesion to UMR-106 Cells After 90 Minutes**

| Concentration (μM) | % of Control (N=6) COMPOUND #1 | COMPOUND #2 | COMPOUND #3 |
|---|---|---|---|
| 5 | 201±6* | 363±17* | 268±14* |
| 12.5 | 404±9* | 293±13* | 300±8* |
| 25 | 262±5* | 316±11* | ND |
| 50 | 24±5* | 30±5* | 30±3* |

Control = 100±12 (18,150 DPM/mg protein);* $p<0.0001$;ND = not determined

**Table 12c. The Effect of Amine-carboxyboranes on J774.A1 Mouse Macrophage Adhesion to UMR-106 Cells After 2 Hours**

| Concentration (μM) | % of Control (N=6) COMPOUND #1 | COMPOUND #2 | COMPOUND #3 |
|---|---|---|---|
| 5 | 98±10 | 106±5 | 105±7 |
| 12.5 | 73±8* | 91±6 | 70±5* |
| 25 | 71±7* | 67±8* | 63±6* |
| 50 | 43±6* | 55±6* | 48±4* |

Control=100±13(13,900DPM/mg protein);*p = <0.0001

**Table 12d The Effects of Amine-carboxyboranes on J774.A1 Mouse Macrophage Adhesion to UMR-106 Cells After 5 Hours**

| Concentration (μM) | % of Control (N=6) COMPOUND #1 | COMPOUND #2 | COMPOUND #3 |
|---|---|---|---|
| 5 | 98±6 | 113±9 | 112±7 |
| 12.5 | 69±5* | 118±8 | 63±6* |
| 25 | 71±6* | 67±5* | 62±7* |
| 50 | 43±4* | 55±5* | 47±4* |

Control = 100±7% (6950 DPM/mg protein); * p<0.0001.

### Discussion

The destructive phase of bone osteoporosis is initiated by cytokines which are chemical cell communicators released by invading macrophages after being stimulated. Early bone resorption effects are evoked by TNFα whereas later effects are regulated by IL-1 and then IL-6 and IL-8. Bone resorption is divided into two phases: phase I involves inorganic demineralization of cortical bone which is a process initiated by influxing macrophages, PMNs, fibroblasts and osteoclasts, etc. and phase II is the organic phase where the bone matrix collagen is digested by proteolytic and hydrolytic enzymes to release hydroproline. Drug therapy should involve blocking the early resorption events as well as the acceleration of the reconstruction of bone.

In vivo studies with the amine-carboxyboranes at 8 mg/kg/day suggest that the calcium uptake and increase in bone collagen deposition had occurred improving the tensile strength of the bone after 14 days treatment[2]. The present studies have demonstrated that amine-carboxyboranes block TNFα and IL-1 release from the types of cells which invade the bone surface to begin the resorption process. Further, these studies showed that these agents also block early adhesion of these migratory cells to the bone cells from 90 min. to 5 hr. and macrophage adhesion is blocked at 2 and 5 hr. at higher concentration of the agents. Blockage of the adhesion of these cell to the bone surface should reduce the hydrolytic and proteolytic enzymes as well as the release of cytokines into the area of the bone surface. Previous studies had demonstrated that the amine-carboxyboranes reduce the activity of macrophages lysosomal and proteolytic enzyme activites with IC-50 values ~$10^{-6}$ M.[1]. Furthermore the amine-carboxyboranes at 8

mg/kg/day were shown in vivo to block PMNs and macrophage infiltration into inflammation sites, i.e. implanted sponges[3,4]. Since bone has high affinity receptors for TNFα and IL-1 as well as other cytokines, the current studies suggest that the cytokine receptors are blocked by the amine-carboxyboranes in a range of 12.5-50 μM.

Whether the compounds are able to displace labeled cytokine from the high affinity receptors because they are agonists or antagonists can not be determined by this type of study. Nevertheless, the blockage of the cytokine receptors positively correlated with certain cellular biochemical events linked with inflammation and bone resorption. For example, the agents effectively blocked bone acid and alkaline phosphatase activities as well as cyclo-oxygenase activity when TNFα binding was maximally reduced by the agents. This 90 min. period also correlated with a reduction on calcium uptake by the bone cells and the lowest level of calcitonin binding to its high affinity receptor on bone cells and of 1,25-dihydro-vitamin-$D_3$ to its nuclear high affinity receptor.

At a later time of 5 hr., the metabolic events reverse themselves. This is at a time when IL-1 high affinity receptor binding is maximally blocked by the amine-carboxyboranes and this time period correlated with the agents inhibiting NAG activity and 5'-lipoxygenase activities as well as reducting the calcitonin high affinity receptor binding, but the 1,25-dihydro-vitamin-$D_3$ high affinity receptor binding was elevated in the presence of the agents. This 5 hr. time period correlated with the elevated uptake of calcium into bone as well the increased incorporation of proline into bone collagen and possiblly non-collagen protein at the lower concentrations of the agents. Inhibition of IL-8 high affinity receptor by the agents occurred at a later time which positively correlated with continued calcium uptake and proline incorporation into protein between 8 and 24 hrs.

Whereas the studies were not conclusive, they did indicate that the early stages of bone resorption with tissue destruction may be regulated by TNFα which can be blocked by the amine-carboxyboranes. Later events regulated by IL-1 could be blocked by the agents by suppressing the high affinity binding activity leading to restoration of dense bone. These finding did support the in vivo studies with these compound in rats for 14 days and do explain why the bone restoration stage was achieved so quickly by the amine-carboxyboranes.

### References:

1. I.H. Hall, K.G. Rajendran, S.Y. Chen, O.T. Wong, A. Sood, B.F. Spielvogel, Arch Pharm (Weinheim) **1995**, 328, 39-44.
2. K.G. Rajendran, S.Y. Chen, A. Sood, B.F. Spielvogel, I.H. Hall. Biomed. Pharmacotherapy. **1995**, in press.
3. I.H. Hall, S.Y. Chen, K.G Rajendran, A. Sood, B.F. Spielvogel, J. Shih, Environ. Health Perspect **1994**, 102 (Suppl 3), 21-30.
4. I.H. Hall, C.O. Starnes, A.T. McPhail, P. Wisian-Neilson, M.K. Das, B.F. Spielvogel, J. Pharm. Sci.**1980**, 69, 1025-1029.
5. F.C. Kull, Jr., Nat Immun. Cell Growth Regul. **1988**, 7, 254-265.
6. F.H. Nielsen, C.D. Hunt, L.M. Mullen, J.R. Hunt, FASEB J., **1987**, 1, 394-397.
7. F.H. Nielsen, L.M. Mullen, S.K. Gallagher, J. Trace Elements Exp. Med. **1990**, 3, 45-54.
8. B. Peterofsky, Arch Biochem. Biophys. **1972**, 152, 318-325.
9. P.S. Chen, T.Y. Toribara, and H. Warner,. Anal. Chem.**1956**, *28*, 1756-1758.

10. H. Tulberg-Reinert, A.F.Hefti, Agents and Actions **1977**, 32, 321-332.
11. R.V. Tomlinson, R.V. Ringold, M.C. Oureshi, E. Forchieli, Biochem. Biophys. Res. Commun. **1972**, *46, 552-558..*
12 M. Glatt, H. Kalin, K. Wagner, K. Brune, Agents Actions **1977**, 7, 321-334.
13. D.L. Flynn, T.R. Belliotti, A.M. Boctor, D.T. Connor, C.R. Kostlan, D.E.,Nies, D.F. Ortwine, D.J. Schrier, J.C. Sircar, J. Med. Chem. **1991**, 34, 518-525.
14. D.L. Flynn, T.Capiris, W.J. Cetenko, D.T. Connor, R.D. Dyer, C.R. Kostlan, D.E. Nies, D.J. Schrier, J.C. Sirar, J. Med. Chem., **1990**, *33*,· 2070-2072.
15. I.H. Hall, R. Simlot, C. Oswald, A.R.K. Murthy, H. ElSourady, J. Chapman Jr., Acta Pharm. Nord. **1990**, 2, 387-400.
16. Y.H. Chin, V. Falanga, J.P.Cai, J. Invest. Dermatol. 1990, 95, 29S-31S

**Received: November 7, 1995 - Accepted: December 7, 1995 - Received in revised camera-ready format: January 9, 1996**

